# RNA-Processing Protein TDP-43 Regulates FOXO-Dependent Protein Quality Control in Stress Response

**DOI:** 10.1371/journal.pgen.1004693

**Published:** 2014-10-16

**Authors:** Tao Zhang, Gerard Baldie, Goran Periz, Jiou Wang

**Affiliations:** 1 **1** Department of Biochemistry and Molecular Biology, Bloomberg School of Public Health, The Johns Hopkins University, Baltimore, Maryland, United States of America, **2** Department of Neuroscience, School of Medicine, The Johns Hopkins University, Baltimore, Maryland, United States of America; The University of North Carolina at Chapel Hill, United States of America

## Abstract

Protein homeostasis is critical for cell survival and functions during stress and is regulated at both RNA and protein levels. However, how the cell integrates RNA-processing programs with post-translational protein quality control systems is unknown. Transactive response DNA-binding protein (TARDBP/TDP-43) is an RNA-processing protein that is involved in the pathogenesis of major neurodegenerative diseases, including amyotrophic lateral sclerosis (ALS) and frontotemporal dementia (FTD). Here, we report a conserved role for TDP-43, from *C. elegans* to mammals, in the regulation of protein clearance via activation of FOXO transcription factors. In response to proteotoxic insults, TDP-43 redistributes from the nucleus to the cytoplasm, promoting nuclear translocation of FOXOs and relieving an inhibition of FOXO activity in the nucleus. The interaction between TDP-43 and the FOXO pathway in mammalian cells is mediated by their competitive binding to 14-3-3 proteins. Consistent with FOXO-dependent protein quality control, TDP-43 regulates the levels of misfolded proteins. Therefore, TDP-43 mediates stress responses and couples the regulation of RNA metabolism and protein quality control in a FOXO-dependent manner. The results suggest that compromising the function of TDP-43 in regulating protein homeostasis may contribute to the pathogenesis of related neurodegenerative diseases.

## Author Summary

TDP-43 is linked to pathogenesis of major neurodegenerative diseases, including amyotrophic lateral sclerosis (ALS) and frontotemporal dementia (FTD). How TDP-43 contributes to the development of these degenerative diseases remains unsolved, and the full range of TDP-43 functions has yet to be established. In the present study, we explored a conversed function of TDP-43 in regulating protein homeostasis from *C. elegans* to mammals. Under conditions of stress, TDP-43 translocates from the nucleus to the cytoplasm, competes with FOXO transcription factors for binding to 14-3-3 proteins, and releases FOXO for nuclear translocation and activation. These data are consistent with the ability of TDP-43 to regulate protein aggregation. Together the results provide important insight into the role of TDP-43 in stress responses and disease mechanisms. Since chronic stress is associated with neurodegenerative diseases, the TDP-43 switch could be kept in overdrive mode in these disorders, with its capacity to buffer further stress and maintain protein homeostasis being compromised. This mechanism also suggests that other RNA-processing proteins that exhibit similar stress-induced behavior may be coupled to other cellular pathways to provide coordinated reprogramming in stress responses.

## Introduction

A defining feature of all living cells is the ability to adapt to stress stimuli. This adaptive response is particularly important for maintaining protein homeostasis, which is critical for cellular functions. The cell employs a variety of protein quality control mechanisms in an effort to maintain the integrity of the proteome, including those regulating protein synthesis and degradation. The regulation of protein synthesis occurs at multiple levels, including transcription [1], RNA processing [2], and translation initiation [3]. Global attenuation of protein synthesis is often part of stress responses, and RNAs and RNA-processing proteins are central players in this adaptation [4]. Meanwhile, coordinated protein quality control systems are activated to enhance the degradation of damaged proteins. For instance, endoplasmic reticulum (ER) stress activates the signal transduction pathway known as the unfolded protein response (UPR), which coordinates a general translational attenuation and a specific induction of quality control proteins, including molecular chaperones, in order to improve protein folding in the ER lumen [5]. However, the coordination between these distinct stress responses is not completely understood and may involve connected regulation at both RNA and protein levels.

TAR-DNA binding protein (TDP-43) is an RNA-binding protein that has been suggested to play a major role in the pathogenesis of amyotrophic lateral sclerosis (ALS) and frontotemporal dementia (FTD) [6]–[34]. Bearing features of a heterogeneous nuclear ribonucleoprotein (hnRNP), TDP-43 has well-characterized RNA-processing functions [35]–[37]. TDP-43 has been shown to regulate transcription [38], [39], RNA splicing [40], [41], mRNA stability [42], [43], and microRNA processing [44]. An increasing number of RNA-binding proteins have been implicated in ALS/FTD and related neurodegenerative diseases [45], including FUS (fused in sarcoma) [46], [47], hnRNPA2B1, and hnRNPA1 [48]. As is true for TDP-43, a pathological feature of these RNA-binding proteins is the formation of proteinaceous inclusions in patients' tissues. Another feature shared by these RNA-binding proteins is their redistribution during stress. Although primarily nuclear, they can be found in stress granules after diverse stimuli [48]–[54]. Although the role of TDP-43 in RNA processing is well established, the full range of TDP-43 function has yet to be understood. Recently, the ortholog of TDP-43 in *C. elegans*, TDP-1, was shown to negatively regulate proteotoxicity associated with protein misfolding, suggesting that the nematode protein plays a role in the regulation of protein homeostasis [55], [56]. This observation raises a question as to whether the RNA-processing function of TDP-43 is directly coupled to its ability to regulate protein homeostasis.

Despite extensive study of the complex pathways responsible for cellular stress responses, how the cell coordinates these different adaptive programs is not yet completely understood. In particular, the exact mechanisms by which RNA processing coordinates with other aspects of stress responses to maintain protein homeostasis remain unclear. Here, we present a mechanistic pathway through which TDP-43 couples RNA processing with active protein quality control during stress. Our results show that TDP-43 regulates the activities of FOXO transcription factors, which are orthologous to *C. elegans* DAF-16 and mediate expression of genes involved in longevity, stress resistance, and protein quality control [57]. The activation of FOXOs is switched on when TDP-43 responds to differential stress signals, undergoes nucleocytoplasmic translocation, and reconfigures its interacting partners. We propose that the regulation of FOXO by TDP-43 represents an important scheme for the cell to efficiently maintain protein homeostasis by exerting control at both the RNA and protein levels; compromising the function of this pathway may contribute to the pathogenesis of TDP-43-related diseases.

## Results

### *C. elegans* TDP-1 regulates lifespan and proteotoxicity through DAF-16

**Figure 1:**
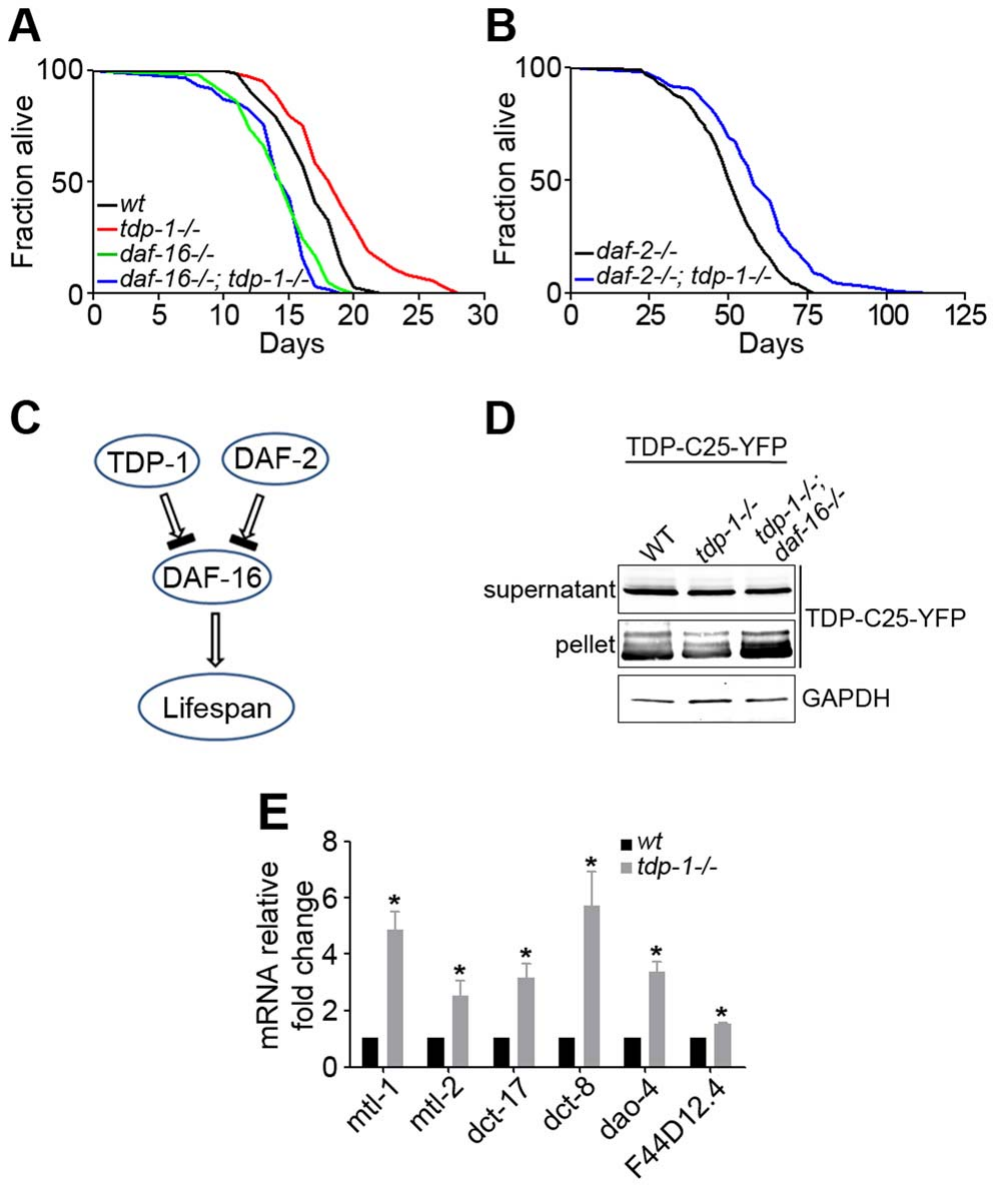
Loss of function of TDP-1 extends the lifespan and reduces protein aggregation in *C. elegans* in a DAF-16-dependent manner. (A) Survival curves of wild-type (black) and *tdp-1(ok803lf)* loss-of-function mutant (red) demonstrate a significant lifespan extension. n>100, *p*<0.001 The lifespan-extending effect of *tdp-1(ok803lf)* is blocked by a loss of function of DAF-16 in *daf-16(mu86lf)*, as shown by the survival curves (green and blue). n>100, *p*<0.001. (B) Survival curves of *daf-2(e1370lf)* loss-of-function mutant alone (black) and the *daf-2(e1370lf);tdp-1(ok803lf)* double mutant (blue) show a further lifespan extension with the loss of TDP-1. n>100, *p*<0.001. (C) Schematic diagram of the genetic pathway for TDP-1 regulation of the lifespan in relation to the DAF-2–DAF-16 pathway. (D) Loss of *daf-16* abolished the reduction of protein aggregation conferred by loss of function of TDP-1. Transgenic TDP-C25-YFP expressed in *C. elegans* neurons was fractionated in to soluble supernatant and insoluble pellet before western blot analysis using a YFP antibody. The effects of single mutant *tdp-1(ok803lf)* and double mutant *tdp-1(ok803lf); daf-16(mu86lf)* were compared. (E) Quantitative measurement of mRNA levels of select DAF-16 targets genes in wild-type and *tdp-1(ok803lf) C. elegans*. n>6, *p<0.001; error bars represent SEM.

*C. elegans* lacking its sole TDP-43 ortholog, TDP-1, lives longer than wild-type (WT) controls (Figure 1A) [55], [56], and the underlying mechanism is not understood. The insulin and insulin-like growth factor (IGF) pathway is an evolutionarily conserved regulator of longevity from *C. elegans* to humans [58]. Reduced function of the insulin/IGF-1 receptor, DAF-2, significantly extends the lifespan by activating DAF-16, a transcription factor that controls the expression of aging-related and stress-resistance genes [59]. To determine whether TDP-1 functions in the DAF-2–DAF-16 pathway, we utilized hypomorphic or null alleles of these genes to perform an epistasis analysis. A double mutant, *tdp-1(ok803lf);daf-2(e1370lf)*, exhibited a longer lifespan than did *daf-2(e1370lf)* alone (Figure 1B). There are further genetic interactions between the two genes on other phenotypes; the *tdp-1;daf-2* double mutant had improved egg-laying and locomotion compared with the *daf-2* mutant alone (Figure S1). These data suggest that TDP-1 acts in a pathway parallel to that of DAF-2 to influence longevity, although a crosstalk between TDP-1 and DAF-2 may still be possible. However, another double mutant, *tdp-1(ok803lf);daf-16(mu86lf)*, completely abolished the longevity effect of *tdp-1(ok803lf)* alone (Figure 1A) [55], indicating that there is a genetic link between TDP-1 and DAF-16, in which DAF-16 lies downstream of TDP-1 in the regulation of lifespan (Figure 1C).

Correlating with increased lifespan in *C. elegans* lacking TDP-1, the mutant also shows enhanced clearance of misfolded proteins [55], [56]. Consistently, we found that the *tdp-1(ok803lf)* mutant reduced the aggregation of TDP-C25-YFP, a misfolded protein reporter expressed in *C. elegans neurons* (Figure 1D). To test whether DAF-16 is downstream of TDP-1, we generated a strain expressing TDP-C25-YFP and harboring the double mutant, *tdp-1(ok803lf);daf-16(mu86lf)*. The *daf-16* mutant reversed the reduction of protein aggregation conferred by the *tdp-1* mutant (Figure 1D). This data suggests that TDP-1 regulates proteotoxicity via DAF-16.

Since DAF-16 is a transcription factor, we asked whether TDP-1 influences the expression of DAF-16's transcriptional targets. We performed quantitative RT-PCR to measure the mRNA levels of a panel of known DAF-16 target genes in *tdp-1(ok803lf)* mutants and WT controls. These DAF-16-regulated genes included stress-resistance genes such as the metallothioneins *mtl-1* and *mtl-2* as well as uncharacterized genes *dao-4*, *dct-8*, and *dct-17*. The results indicated that most of the tested DAF-16 target genes are significantly up-regulated in *tdp-1(ok803lf)* mutant *C. elegans* (Figure 1E).

Next we asked whether this up-regulation upon loss of TDP-1 is specific to DAF-16 target genes. Although the transcriptional profiles of *tdp-1(ok803lf)* mutants and WT controls indicates that there are more genes down-regulated than up-regulated in *tdp-1(ok803lf)* mutants (Figure S2A) [55], quantitative RT-PCR analysis shows that DAF-16 targets are specifically up-regulated (Figure S2B). Taken together, these data demonstrate that loss of *tdp-1* produces a specific up-regulation of DAF-16 transcription factor activity.

### *C. elegans* TDP-1 switches its localization in response to proteotoxic stress

Since DAF-16 is a major transcription factor of stress-resistance genes [59], and mammalian TDP-43 undergoes stress-induced localization changes [49]–[51], we hypothesized that *C. elegans* TDP-1 is involved in stress signaling. To determine whether TDP-1 undergoes stress-induced changes in neurons, we generated transgenic strains that expressed YFP-tagged TDP-1 under the control of the pan-neuronal *snb*-1 promoter. These animals exhibited severe locomotor defects similar to those that we have previously noted in transgenic *C. elegans* expressing human TDP-43 driven by the same neuronal promoter [60].

**Figure 2:**
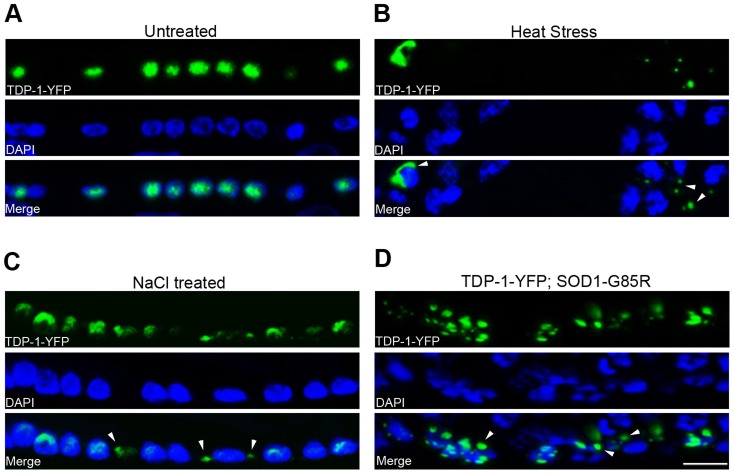
*C. elegans* TDP-1 forms cytoplasmic granules in neurons under proteotoxic stress. Transgenic *C. elegans* expressing TDP-1-YFP under a neuronal promoter were treated with the indicated proteotoxic stressors prior to fixation, DAPI staining, and visualization by florescence microscopy. (A) In untreated *C. elegans*, TDP-1-YFP shows primarily nuclear localization. Ventral cord neurons are shown. (B) When heat-stressed at 28°C for 16 h, TDP-1-YFP shows cytoplasmic localization and forms granular structures. Nerve ring neurons are shown. (C) When treated with 0.4 M NaCl for 24 h, TDP-1-YFP also translocates to the cytoplasm and forms granules. Ventral cord neurons are shown. (D) When crossed to a transgenic strain stably expressing the ALS mutant SOD1-G85R in neurons, a subset of animals shows cytoplasmic translocation and granule formation of TDP-1-YFP. Arrowheads point to stress-induced TDP-1-YFP granules. Scale bar: 5 µM.

Since protein quality control is involved in the regulation of both lifespan and neurodegeneration, we investigated whether TDP-1 responds to proteotoxic stress. First, we observed that neuronal TDP-1 responds to heat shock stress, which is known to increase misfolded proteins. When the transgenic TDP-1-YFP strain was grown on solid or liquid medium at 20°C, the TDP-1-YFP protein was localized to neuronal nuclei (Figure 2A). However, when the strain was subjected to heat shock stress at 28°C for 16 hours, TDP-1-YFP migrated to the cytoplasm, and in a subset of neurons, the protein formed granular structures (Figure 2B). Next, we tested the effects of hypertonic stress, which has been shown to induce molecular crowding and protein damage [61]–[64]. When the strain was treated with 0.4 M NaCl in liquid medium, we again observed the nucleocytoplasmic translocation of TDP-1 and formation of granular structures (Figure 2C). To test the response of TDP-1 in a setting directly relevant to proteotoxicity-related neurodegeneration, we crossed the stable transgenic TDP-1-YFP *C. elegans* strain into a *C. elegans* model of ALS expressing human SOD1 with the G85R mutation. The SOD1-G85R mutant has a high propensity to misfold and aggregate in this model system [65]. In the double-transgenic strain expressing both TDP-1-YFP and SOD1-G85R, but not the single-transgenic strain, we observed a switch in localization of TDP-1-YFP from the nucleus to the cytoplasm, where it was localized to punctate granules similar to those observed under heat shock and hypertonic stress (Figure 2D). These TDP-1 puncta could be distinct RNA granules; alternatively, the presence of misfolded proteins, such as SOD1-G85R, may seed the aggregation of TDP-1. Taken together, these results demonstrate that TDP-1 responds to different types of proteotoxic stresses, suggesting that the regulation of lifespan by TDP-1 involves its function in stress signaling.

### Regulation of FOXO activities by TDP-43 in human cells

**Figure 3:**
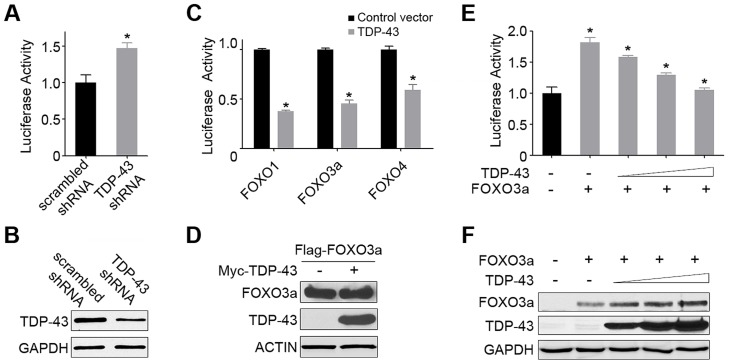
TDP-43 negatively regulates the transcriptional activity of FOXOs in mammalian cells. (A) The FOXO transcriptional activity is significantly increased by the knockdown of endogenous TDP-43 in HEK293T cells. Cells were transfected with an FHRE-Luc reporter, a *Renilla* luciferase control, a FOXO3a-expressing construct, and an shRNA construct against endogenous TDP-43 or a scrambled control shRNA. Cell lysates were subjected to dual luciferase assays, and the ratio of firefly *to Renilla* luciferase activity was used to measure the FOXO transcriptional activity. (B) The knockdown of TDP-43 in HEK293T cells was confirmed by western blotting. GAPDH was used as a loading control. (C) The FOXO transcriptional activity was inhibited by the expression of TDP-43 in HEK293T cells. Cells were transfected with the FHRE-Luc reporter, the *Renilla* luciferase control, and one of the FOXO family members (FOXO1, FOXO3a, or FOXO4) for measurement of their respective activities in the presence or absence of Myc-TDP-43. (D) The protein levels of FOXOs, as represented by FOXO3a, were not reduced with the expression of TDP-43, as shown by western blotting. (E) The FOXO transcriptional activity, as represented by FOXO3a, was inhibited by TDP-43 in a dose-dependent manner. Cells were transfected as described above but with increasing amounts TDP-43-expressing constructs (0, 50, 100, or 200 ng DNA per well on 24-well plates). (F) The protein levels of FOXOs, as represented by FOXO3a, were not reduced by increasing levels of TDP-43, as shown by western blotting. n>3, *p<0.05; error bars represent SEM.

Next, we asked whether the observed regulation of DAF-16 by TDP-1 is conserved from *C. elegans* to humans. DAF-16 is the sole *C. elegans* ortholog of four mammalian FOXO members (FOXO1, FOXO3a, FOXO4, and FOXO6), with the first three showing a high degree of structural and regulatory similarity [66]. To determine whether TDP-43 regulates the transcriptional activity of FOXOs, we used a luciferase reporter under the control of forkhead responsive elements (FHRE-Luc) to measure the FOXO transcriptional activity in HEK293T human embryonic kidney cells. Co-expression of the FHRE-Luc reporter with a FOXO family member significantly boosted the luciferase signal, enhancing the sensitivity for measuring the activity of a particular FOXO transcription factor. Consistent with the up-regulation of *C. elegans* DAF-16 activity by loss of TDP-1, shRNA-mediated knockdown of endogenous TDP-43 in HEK293T cells significantly increased the FOXO transcriptional activity, as indicated by the increase in luciferase activity (Figure 3A–B). Conversely, ectopic expression of TDP-43 markedly decreased the transcriptional activity of all three FOXO family members (Figure 3C–D). Moreover, the effects of TDP-43 on FOXO transcriptional activity were dose-dependent, with increasing levels of TDP-43 causing further suppression of the FHRE-Luc reporter signal. This suppression of FOXO transcriptional activity was not due to a decrease in FOXO protein levels caused by the overexpression of TDP-43 (Figure 3E–F). Overexpression of TDP-43 alone did not significantly change the luciferase activity of the FHRE-Luc reporter, reflecting the fact that the low endogenous level of FOXOs is not sufficient for the assay (Figure S3) [67]. Given the primarily nuclear localization of TDP-43, these results suggest an inhibition of FOXO transcriptional activity by TDP-43 in the nucleus. Taken together, these results establish a regulation of FOXO by TDP-43 that is conserved from *C. elegans* to humans.

### TDP-43 localization is determined by the type and strength of stress signals

**Figure 4:**
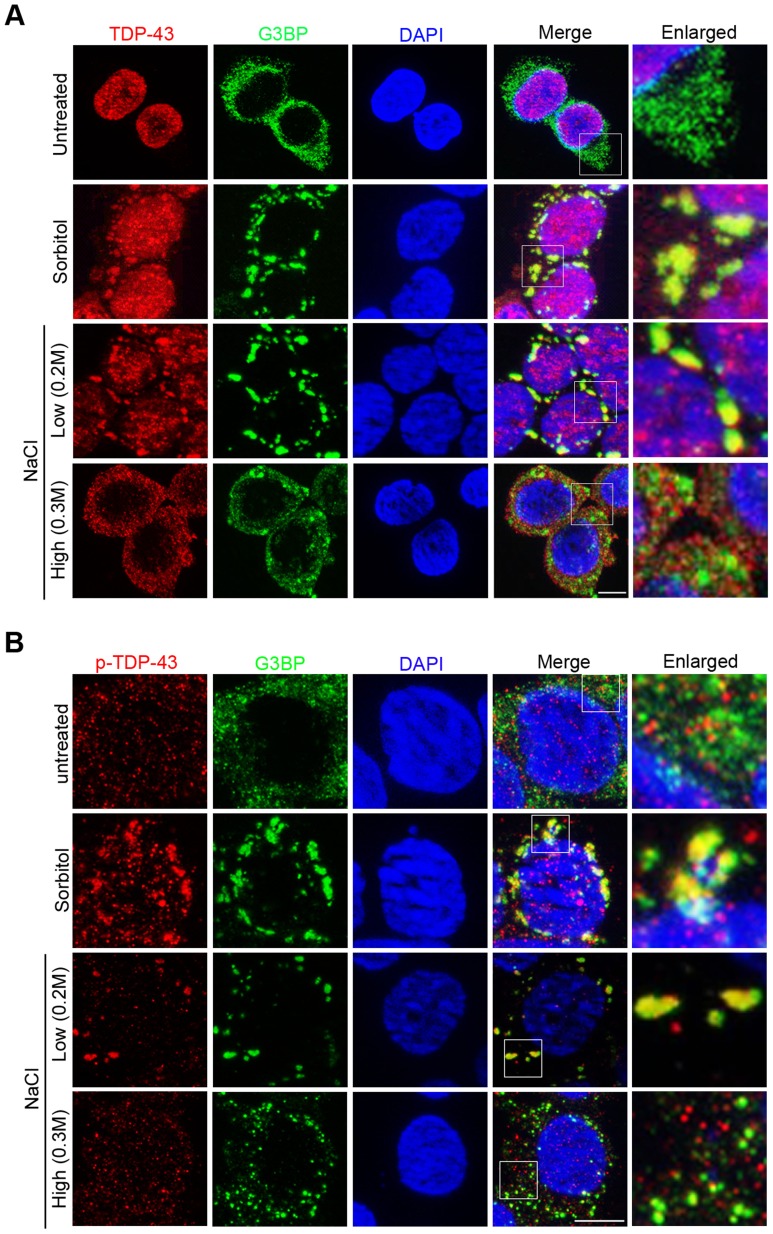
Hypertonic stress with NaCl induces different forms of cytoplasmic translocation and granule formation of TDP-43 in a concentration-dependent manner. (A) The localization patterns of endogenous TDP-43 under different stress conditions. In unstressed HEK293T cells, total TDP-43 is predominantly nuclear (top panel). With sorbitol and low NaCl (0.2M) stress, TDP-43 translocates to the cytoplasm and colocalizes with the stress granule marker G3BP (middle panels). With high NaCl (0.3M) stress, TDP-43 translocates to the cytoplasm but forms smaller granules that do not colocalize with G3BP (bottom panel). Scale bar: 5 µM. (B) Phosphorylated TDP-43 (p-TDP-43) is primarily localized to the cytoplasm. With sorbitol and low NaCl (0.2M) stress, p-TDP-43 colocalizes with G3BP. With high NaCl (0.3M) stress, p-TDP-43 does not colocalize with G3BP. Scale bar: 5 µM.

Since NaCl treatment induced the nucleocytoplasmic translocation and granule formation of *C. elegans* TDP-1, we asked whether this response to hypertonic stress is evolutionarily conserved, by examining the effect of NaCl treatment on human TDP-43 in HEK293T cells through immunofluorescent staining. Treatment of HEK293T cells with 0.2 M NaCl induced the translocation of TDP-43 from the nucleus to the cytoplasm, where it co-localized with the stress granule marker Ras GTPase-activating protein-binding protein 1 (G3BP) (Pearson's correlation coefficient>0.9) (Figure 4A). The redistribution of TDP-43 to cytoplasmic G3BP-positive structures with treatment of 0.2 M NaCl was similar to that observed after treatment with sorbitol, a known osmotic stressor and well-established inducer of stress granule formation [68]. To examine the dynamic redistribution of TDP-43 during hypertonic stress, we stained for TDP-43 after incubating HEK293T cells with 0.2M NaCl for various lengths of time (Figure S4). Changes in TDP-43 were observed within 15 min of NaCl treatment, with TDP-43 forming punctate structures in the nucleus. By 2 h, TDP-43 was observed in the cytoplasm in large stress granules co-localizing with G3BP.

Interestingly, we found that NaCl-stress-induced patterns of TDP-43 translocation and sequestration varied in a concentration-dependent manner. When HEK293T cells were treated with 0.3 M NaCl, TDP-43 underwent cytoplasmic translocation but was recruited to a previously undescribed, smaller type of granules that did not co-localize with G3BP (Pearson's correlation coefficient <0.4) (Figure 4A). We also examined the dynamic redistribution of TDP-43 with 0.3 M NaCl treatment (Figure S5). TDP-43 formed punctate granules in nucleus within 15 minutes, and by 30 minutes TDP-43 redistributed to the cytoplasm forming this type of small granules.

Next we explored whether the translocation of TDP-43 is a reversible process. After a 3-h treatment with sorbitol or NaCl, the stressors were washed off, and the cells were kept in normal medium. After 24 h, TDP-43 was completely translocated back to the nucleus, although G3BP-positive stress granules remained in the cytoplasm (Figure S6). These results indicate that TDP-43 responds to various stresses in a dynamic and reversible manner that is not always associated with stress granules.

Since redistribution of proteins in the cell is often associated with post-translational modifications, we examined how the phosphorylation of TDP-43 correlates with the changes in its localization induced by hypertonic stress. We used a phospo-TDP-43 Ser409/410-specific antibody to detect the phosphorylated TDP-43 in HEK293T cells by immunofluorescence microscopy (Figure 4B). In untreated HEK293T cells, unlike the unmodified TDP-43, the phosphorylated protein appeared in both the nucleus and cytoplasm. As has previously been observed for sorbitol [49], low hypertonic stress (0.2M NaCl) treatment resulted in the majority of the phosphorylated TDP-43 co-localizing with G3BP-positive stress granules in the cytoplasm (Pearson's correlation coefficient>0.9). In contrast, when cells were exposed to high hypertonic stress (0.3M NaCl), the phosphorylated TDP-43 was localized differently and produced an appearance similar to that of untreated cells: The phosphorylated TDP-43 was distributed throughout both the nucleus and cytoplasm and did not colocalize with the G3BP-positive stress granules (Pearson's correlation coefficient <0.4). These results indicate that although cytoplasmic translocation is a consistent feature of TDP-43 during stress responses, its localization patterns are determined by the type and strength of the stress signals.

### TDP-43 controls nuclear translocation of FOXOs through competitive binding to 14-3-3 proteins

To understand how TDP-43 could regulate the activity of FOXOs during stress responses, we investigated the protein interactions that link these two proteins. We co-transfected HEK293T cells with tagged versions of the TDP-43 and FOXO3a proteins and performed co-immunoprecipitation assays. Immunoprecipitation assays using TDP-43 as bait failed to pull down FOXO3a (Figure S7), indicating that there is no physical interaction between TDP-43 and FOXO3a.

**Figure 5:**
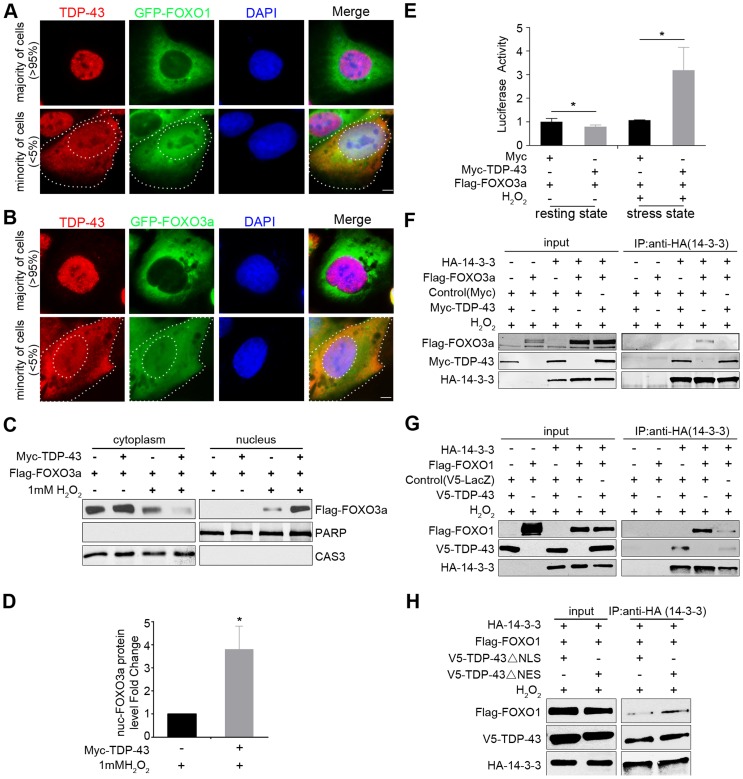
Cytoplasmic translocation of TDP-43 drives the nuclear translocation of FOXOs, and TDP-43 competes with FOXOs for binding to 14-3-3 sigma. (A–B) The localization patterns of endogenous TDP-43 (red) and stably expressed GFP-FOXO1 (A) or GFP-FOXO3a (B) (green) in U2OS cell lines. The majority of the cells (>95%) show predominantly nuclear TDP-43 with cytoplasmic FOXOs. A minority of the cells (<5%) show cytoplasmic TDP-43 staining, while FOXOs translocate from cytoplasm to nucleus. Scale bar: 5 µM. (C–D) Fractionation assays show stress-induced cytoplasmic translocation of TDP-43 drives the nuclear translocation of FOXOs. HEK293T cells were transfected with Flag-FOXO3a with or without Myc-TDP-43. Only when cells were both transfected with TDP-43 and subjected to 1 mM H_2_O_2_ stress, there was a significant increase in the nuclear fraction of FOXO3a. PARP1 and Caspase 3 are shown as nuclear and cytoplasmic markers, respectively. n>3, * *p*<0.05; error bars represent SEM. (E) When HEK293 cells are in the resting state, the FOXO3a transcriptional activity is significantly inhibited by ectopically expressed TDP-43. n>3, *p<0.05. When HEK293 cells are exposed to 1 mM H_2_O_2_ stress, the FOXO3a transcriptional activity is significantly increased by the ectopically expressed TDP-43. n>3, *p<0.05; error bars represent SEM. (F,G) HEK293T cells were co-transfected with different combinations of HA-14-3-3σ, Myc-TDP-43, and either Flag-FOXO3a (F) or Flag-FOXO1 (G) as indicated in the presence of 1 mM H_2_O_2_ stress. Co-immunoprecipitation was performed using antibodies against HA-14-3-3σ for the pulldown and the indicated proteins for the western blotting. Increased amounts of TDP-43 interrupted the interaction between FoxO3a and 14-3-3σ (F). TDP-43 interrupted the interaction between FoxO1 and 14-3-3σ (G). (H) HEK293T cells were co-transfected with different combinations of HA-14-3-3σ, V5-TDP-43 variants, and Flag-FOXO1 as indicated in the presence of 1 mM H_2_O_2_ stress. ΔNLS TDP-43 was more effective than ΔNES in competing with FOXO3a for the interaction with 14-3-3σ.

Next we investigated whether TDP-43 and FOXOs are linked in their localization changes during stress responses. To easily visualize the localizations of FOXO proteins, we utilized two U2OS cell lines that stably expressed GFP-FOXO1 or GFP-FOXO3a. Interestingly, we observed a strong mutual exclusion in the nucleocytoplasmic compartmentalization of TDP-43 and FOXO proteins (Figure 5A–B). Under normal culture conditions, endogenous TDP-43 was primarily localized in the nucleus, and only less than 5% of cells had a significant fraction of the TDP-43 in the cytoplasm, with the percentage increasing with stress. When TDP-43 was in the nucleus, the GFP-tagged FOXOs were almost invariably in the cytoplasm. When TDP-43 was cytoplasmic, the majority of the cells showed translocation of the FOXO proteins to the nucleus. Under oxidative stress induced by H_2_O_2_ treatment, cytoplasmic translocation of TDP-43 and nuclear translocation of FOXOs both increased, and their exclusive spatial correlation was maintained.

We then asked whether the cytoplasmic translocation of TDP-43 specifically drives the nuclear translocation of FOXO proteins as a stress response. To address this question, we performed cellular fractionation assays using HEK293T cells co-transfected with tagged versions of the TDP-43 and FOXO3a proteins and treated with hydrogen peroxide, which consistently induces TDP-43 cytoplasmic translocation. Western blotting against a cytoplasmic marker (caspase-3) and a nuclear marker (PARP1) confirmed a clean separation of cytoplasmic and nuclear fractions. The expression of TDP-43 alone, without stress, did not induce any detectable change in the fractionation of FOXO3a. However, with 1 mM H_2_O_2_ treatment, which increases the cytoplasmic fraction of TDP-43, the expression of TDP-43 led to a pronounced shift in FOXO3a from the cytoplasm to the nucleus (Figure 5C–D). These results suggest that the redistribution of TDP-43 from the nucleus to the cytoplasm under stress drives FOXO proteins translocating from the cytoplasm to the nucleus, consistent with the observation of a strong mutual exclusion in the nucleocytoplasmic compartmentalization of TDP-43 and FOXO proteins. To test whether TDP-43 also regulates the function of FOXOs in a stress-dependent manner, we used the FHRE-Luc reporter to measure FOXO transcriptional activity in the absence or presence of TDP-43. In contrast to the unstressed condition in which the expression of TDP-43 significantly suppressed FOXO activities, the TDP-43 expression dramatically increased FOXO3a transcriptional activity under the H_2_O_2_ stress (Figure 5E). Thus TDP-43 plays a pronounced role in stress to promote the nuclear translocation and transcriptional activity of FOXO proteins.

To understand how TDP-43 regulates FOXO localization and activity without a physical interaction between the proteins, we asked whether there is another player that might mediate an indirect association between them. The 14-3-3 family of proteins emerges as a possible candidate because these proteins have been shown to be involved in a multitude of signaling pathways and have a diverse set of binding partners. FOXO1, FOXO3a, and FOXO4 all interact with 14-3-3 proteins [69]–[71], and TDP-43 has been shown to interact with 14-3-3 proteins in an RNA-dependent manner [43]. To determine whether 14-3-3 relays signals from TDP-43 to FOXO, we performed competitive co-immunoprecipitation assays to address whether 14-3-3 partners with TPD-43 and FOXO in mutually exclusive protein complexes (Figure 5F–H). For this purpose, we transfected HEK293T cells with different combinations of Myc-tagged TDP-43, Flag-tagged FOXO3a, and HA-tagged 14-3-3σ. With 1 mM H_2_O_2_ treatment, 14-3-3σ was able to pull down either TDP-43 or FOXO3a when 14-3-3σ was co-transfected with either of the two proteins (Figure 5F). However, when all three proteins were expressed, the level of FOXO3a was greatly reduced in the 14-3-3σ co-immunoprecipitates (Figure 5F). Similar competitive binding to 14-3-3σ was also observed between WT TDP-43 and FOXO1 (Figure 5G). These results demonstrate that TDP-43 and FOXOs compete for binding to 14-3-3.

To further examine the relationships among these three proteins, we studied the effect of mutant TDP-43 proteins lacking the nuclear localization signal (ΔNLS) or the nuclear export signal (ΔNES) on the interaction between14-3-3 and FOXO proteins using the same competitive co-immunoprecipitation assays described above. The ΔNLS and ΔNES mutations enhanced the relative enrichment of cytoplasmic and nuclear TDP-43, respectively (Figure S8). The compartmentalization of the mutants was not exclusive, since residuals of the ΔNLS and ΔNES mutants could be found in the nuclear and cytoplasmic fractions. Nevertheless, the ΔNLS and ΔNES mutants allowed us to address how localization of TDP-43 affects the competitive binding of FOXOs to 14-3-3 proteins. We transfected HEK293T cells with tagged versions of FOXO3a, 14-3-3σ, and ΔNLS or ΔNES TDP-43, and then performed co-immunoprecipitation experiments using 14-3-3 as the bait. The ΔNLS TDP-43 was more effective than ΔNES in interfering with the interaction between 14-3-3σ and FOXO3a, indicating that the cytoplasmic fraction of TDP-43 is capable of dissociating FOXO3a from its binding to 14-3-3. This result suggests that the competitive binding of TDP-43 to 14-3-3 occurs at least in part in the cytoplasm (Figure 5H).

### TDP-43 regulates protein quality control

**Figure 6:**
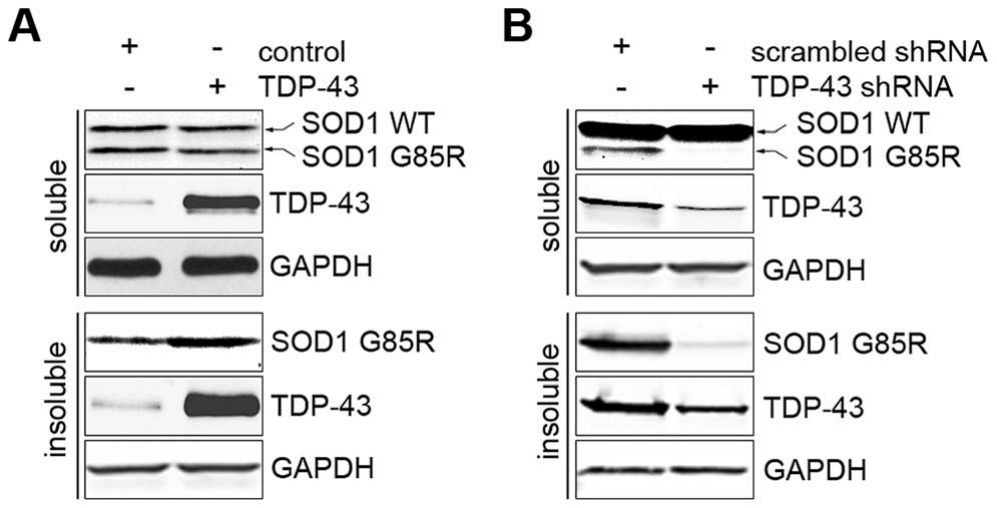
TDP-43 regulates levels of misfolded proteins. (A) Ectopic expression of TDP-43 increases the aggregation formation of SOD1 G85R. HEK293T cells were co-transfected with plasmids expressing SOD1 G85R and WT TDP-43. The cells were detergent-extracted to separate soluble proteins (unaggregated and oligomers) and insoluble aggregates. (B) Knockdown of TDP-43 suppressed the aggregation of SOD1 G85R. HEK293T cells were co-transfected with plasmids expressing SOD1 G85R and shRNA against TDP-43 or a scrambled control. The cells were detergent-extracted as described and subjected to western blotting analysis.

FOXOs have been reported to positively regulate protein quality control systems, including proteasomes and autophagy [72]–[74]. The regulation of FOXOs by TDP-43 suggests that TDP-43 may be a regulator of protein quality control. To explore this possibility, we studied the effects of loss or gain of TDP-43 on protein aggregation using a previously established SOD1 solubility assay [75]. The assay relies on differential detergent extraction to separate insoluble protein aggregates from soluble low-molecular weight monomers and oligomers. The G85R mutant, but not WT SOD1, was found in the insoluble pellet, providing a sensitive reporter for protein aggregation. When TDP-43 was ectopically expressed in HEK293T cells, there was a significant increase in the level of insoluble G85R SOD1 aggregates, with no difference in the soluble level (Figure 6A). However, when TDP-43 was knocked down in HEK293T cells, there was a marked reduction in the insoluble aggregates of G85R SOD1, together with a decrease in the level of soluble mutant SOD1 (Figure 6B). The WT SOD1 protein level was not changed when TDP-43 was knocked down or overexpressed, suggesting that TDP-43 negatively regulates the turnover of misfolded proteins.

## Discussion

In the present study, we have described a conserved signaling pathway in which TDP-43 senses stress and regulates protein quality control. This pathway is mediated, at least in part, by the ability of TDP-43 to regulate FOXO transcription factors. Therefore, TDP-43, a known RNA binding protein, represents a link between protein quality control and RNA metabolism, indicating a novel layer of regulation of protein homeostasis imparted by RNA-processing proteins. We propose that TDP-43 mediates stress responses designed to maintain protein homeostasis by coordinating the attenuation of protein synthesis and the selective enhancement of protein quality control systems. First, the loss of TDP-43 from the nucleus and its localization to stress granules in response to cellular stress constitute an adaptive response to keep target mRNAs from active translation [76]. Second, the cytoplasmic translocation of TDP-43 promotes protein quality control, increasing the removal of misfolded proteins through the regulation of FOXOs described here. Thus, the connection between the two metabolic processes, RNA processing and protein quality control, represents a high-level regulation of protein homeostasis, accomplished through TDP-43 coordination.

**Figure 7:**
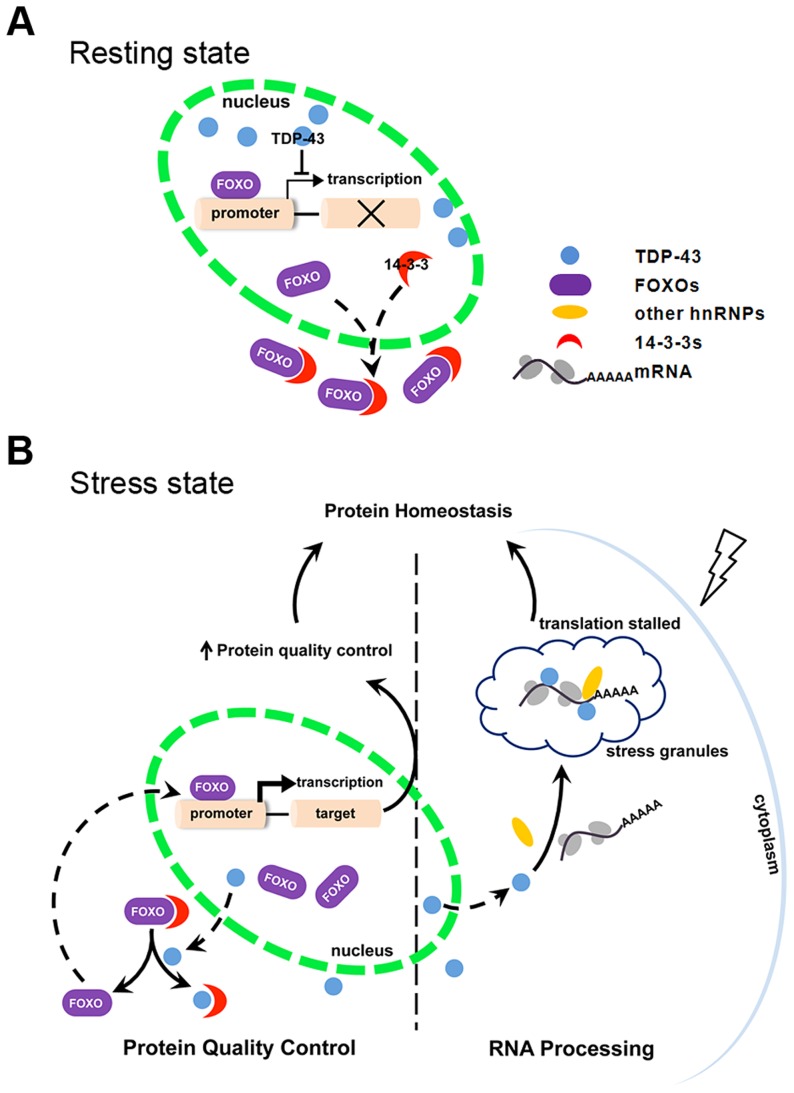
Schematic diagram summarizing the mechanism by which TDP-43 acts as a stress-response switch to regulate protein homeostasis. (A) In the resting state, FOXO is retained significantly in the cytoplasm. TDP-43, which is predominantly nuclear, inhibits nuclear FOXO transcriptional activity. (B) In response to stress, TDP-43 undergoes cytoplasmic translocation and helps maintain protein homeostasis by both recruiting untranslated mRNAs to stress granules and promoting protein quality control. The recruitment of TDP-43 and target mRNAs to cytoplasmic granules contributes to slower translation and reduced protein-folding burden. The competitive binding of TDP-43 to 14-3-3 proteins drives nuclear translocation and activation of FOXOs. Consequently, FOXO-mediated protein quality control is activated.

The regulation of FOXO transcription factor activity by TDP-43 has not been previously described. In this regulation, TDP-43 acts as a stress response switch to control FOXO activities. When the cell is in the resting state, TDP-43 is predominantly nuclear and exerts negative control over the FOXO transcription factors (Figure 7A). Evidence for this model includes TDP-1's negative regulation of DAF-16 transcriptional activity in *C. elegans* (Figure 1) and TDP-43's negative regulation of FOXO transcription activity in mammalian cells (Figure 3). When the cells are exposed to stress, TDP-43 temporarily leaves the nucleus and relaxes its negative control of the FOXO transcription factors. TDP-43 does not appear to influence the levels of FOXO proteins, which are governed by other complex regulations. Instead the increased fraction of cytoplasmic TDP-43 competes with FOXOs for binding to 14-3-3 proteins and drives the nuclear translocation of the FOXOs, further enhancing their transcription activity (Figure 7B).

The regulation of FOXOs by TDP-43 is consistent with the changes we observed in the cell's protein quality control systems. DAF-16, the only ortholog of the FOXO transcription factors in *C. elegans*, promotes longevity by activating the transcription of stress-resistant genes, including molecular chaperones. Accordingly, the loss-of-function TDP-1 mutant has an increased lifespan that is DAF-16-dependent, and it also has reduced levels of misfolded proteins (Figure 1) [55], [56]. In mammalian cells, the activation of FOXO transcription factors, including FOXO1 and FOXO3a, induces autophagy [72]–[74], [77]; the activation of FOXO3a also promotes proteasome activity [73]. In accordance with the negative regulation of FOXOs by nuclear TDP-43, acute reduction of TDP-43 decreases the levels of misfolded and aggregated proteins (Figure 6), suggesting enhanced protein quality control.

The coupling of RNA regulation to protein quality control by TDP-43 may represent a general coordination feature among stress-adaptive programs. There are other RNA-processing proteins that exhibit similar switching behaviors during stress responses. For example, a number of hnRNP proteins translocate to the cytoplasm from the nucleus in response to stress and are sequestered in punctate structures such as stress granules. We propose that, like TDP-43, these RNA-processing proteins function as stress response switchers to maintain cellular homeostasis. The nucleocytoplasmic translocation and sequestration of these RNA-processing proteins into stress granules represent an adaptive loss of function that serves to temporarily curtail protein synthesis.

TDP-43's role in controlling stress response and protein homeostasis may have important implications for neurodegenerative diseases. Rather than a simple gain- or loss-of-function scenario, we propose that a mechanism involving the compromised function of TDP-43 acting as a stress response switch underlies the etiology and pathology of TDP-43-related degenerative diseases. The TDP-43 proteinopathy is characterized by the cytoplasmic accumulation and concomitant nuclear clearance of the non-mutated form of the TDP-43 protein [6]. This common pathology in related neurodegenerative diseases is likely a result of the response to chronic stress. Moreover, the present study suggests that there is a duality in the cellular effects of TDP-43 cytoplasmic translocation and nuclear clearance. As a stress response, this acute reduction in TDP-43 function is protective, defending the cell against proteotoxicity through the pathway delineated above. However, if chronic stress persists, the resulting long-term reduction in TDP-43 function is deleterious. Without a resetting of the switch, the capacity of TDP-43 to buffer further stress would be lost. Also, over-activation of FOXOs promotes senescence or cell death [78], suggesting that the activation of FOXOs by the TDP-43 switch may initiate a built-in program to eliminate over-stressed cells. This duality is analogous to that in ER stress, in which the UPR program has both a protective and a deleterious effect [5].

In conclusion, TDP-43 acts as a stress response switch to regulate RNA and protein metabolism in order to maintain protein homeostasis. The role of TDP-43 in the feedback regulation of the proteotoxic stress response and quality control provides a new perspective for TDP-43-related pathogenesis. Compromise of the stress response by proteins like TDP-43 may be a general mechanism underlying neurodegenerative diseases.

## Materials and Methods

### *C. elegans* and transgenic animals

All *C. elegans* strains are on the N2 Bristol background and cultured under standard conditions at 20°C unless otherwise indicated. To generate the P*snb-1*::TDP-1-YFP(*iwIs53*) strain, a transgene DNA construct was generated by subcloning TDP-1 cDNA with a C-terminal YFP tag into a modified plasmid, pPD30_38 (Fire Lab Vector, Addgene), with the promoter replaced with that of the *snb-1* gene, as previous described [60]. The transgene DNA solution containing 20 ng/µl of the expression construct was injected into hermaphrodite gonads [79], and multiple extrachromosomal lines were established based upon the fluorescent markers. These lines were further treated with 30 µg/ml trimethylpsoralen (Sigma-aldrich) and 300 µJ of 365 nm UV light to screen for integrated lines that stably expressed the transgenes. Each integrated line was backcrossed with the N2 strain at least four times. The P*snb-1*::TDP-C25-YFP(*iwIs22*) strain was reported previously [60]. Some strains were provided by the Caenorhabditis Genetics Center, which is funded by the NIH Office of Research Infrastructure Programs (P40 OD010440).

### *C. elegans* lifespan analysis

The WT N2 *C. elegans* and mutant strains RB929 [*tdp-1(ok803)*], CF1038 [*daf-16(mu86)*], CB1370 [*daf-2(e1370)*], IW417 [*tdp-1(ok803); daf-16(mu86)*], and IW177 [*tdp-1(ok803);daf-2(e1370)*] were cultured under standard conditions at 20°C. Newly laid embryos were synchronized within 2 h and transferred to fresh NGM plates, with 50 embryos per plate. These animals were transferred to new plates every day until they reached the post-reproductive stage and were allowed to age under normal culture conditions. Animals were checked daily and considered dead if they showed no response to probing with a platinum pick. The animals that crawled out of the plate, had vulval burst, or died with internally hatched larvae or “bags of worms” were censored. The day when embryos were synchronized was defined as the first day for lifespan analysis. The lifespan data were analyzed using Prism 5 software.

### Egg-laying and locomotion assays

*C. elegans* were cultured at 20°C until they grow to L4 larval stage. L4 larvae were individually transferred to new plates and cultured at 25°C. Every 24 hours, these animals were transferred to new plates at 25°C until they stopped producing eggs. After transferring, the eggs laid on the plates were counted. For locomotion measurement, L4 larvae grown at 20°C were subject to a thrashing assay. The animals were transferred into M9 buffer (3 mg/ml KH2PO4, 6 mg/ml Na2HPO4, 5 mg/ml NaCl, and 1 mM MgSO4) and allowed to adapt to the buffer for 1 min. Then the rate of body bending or thrashes for the animals was measured, with a thrash being counted when both the head and the tail bend over 45 degrees.

### Quantitative PCR analysis of gene expression

To quantify the expression of specific genes in *C. elegans*, animals were harvested and total RNA was isolated using a phenol-chloroform extraction with TRIzol reagent (Life Technologies), followed by purification with an RNeasy mini kit (Qiagen). A two-step RT-PCR was employed to assess relative changes in transgenic transcripts using an iScript cDNA Synthesis Kit and an IQ SYBR Green kit (Bio-Rad). Fluorescence was measured on a real-time PCR cycler (Bio-Rad), and C_T_ values were analyzed based on standard curves. The worm *gdh-1* gene was used as a control. The sequences of all the primers used are listed in the Table S1.

### Gene expression and knockdown in tissue culture

HEK293T cells were grown in Dulbecco's modified Eagle's medium supplemented with antibiotics and 10% fetal bovine serum. Transient transfection of HEK293T was carried out with Lipofectamine 2000 according to the manufacturer's instructions (Life Technologies). The primers for subcloning TDP-43 cDNA into pRK5-myc vector at *Sal* site and Gateway vector (pDonor-221) are listed in the Table S1.

To knock down the expression of TDP-43, we generated shRNA constructs targeting different regions of the TDP-43 transcript and containing a puromycin resistance gene. The TDP-43 shRNA constructs were cloned by inserting small hairpin oligonucleotides matching TDP-43 mRNA sequences into the pRFP-C-RS plasmid (Origene) using *BamH*I/*Hind*III restriction sites. The sequence of shRNA oligonucleotides (i), (ii), and (iii) as well as a control shRNA-RFP-C-RS are listed in the Table S1. HEK293T cells were plated in 60-mm dishes at a density of 3.5×10^5^ per well, then transfected with the shRNA constructs after 24 h. After 24 h, puromycin was applied at 3 µg/ml to select for positively transfected cells, and cells were harvested at 72 h post-transfection.

### Luciferase reporter assays for FOXO activity

A reporter construct, pGL3-FHRE-Luc was originally from M. Greenberg (Addgene Plasmid 1789), which expresses the firefly luciferase driven by a promoter containing three copies of forkhead responsive elements, was employed to measure FOXO transcription activity [80]. A control reporter construct, pSV40-Renilla (Promega), which provides constitutive expression of *Renilla* luciferase, was used as an internal control. Cells were plated in 24-well plates at a density of 1×10^5^ cells per well, and after 24 h, cells were transfected with expression plasmids of FOXOs, pSV40-Renilla, pGL3-FHRE-luciferase, or TDP-43. At 48 h, luciferase activities were measured by using the Dual-Luciferase Reporter Assay System (Promega) on a Synergy H1 luminometer (Bio-Tek). The experimental firefly luciferase activity was normalized to the control *Renilla* luciferase activity to reflect the FOXO activities.

### Cell stress, immunofluorescent staining, and microscopy

In addition to HEK293T cells, two U2OS stable cells lines expressing FOXO1 or FOXO3a were used (Thermofisher). Cells were plated on coverslips pre-treated with polyethylenimine (Sigma-aldrich), in 6-well plates. Cells were stressed with 0.4 M sorbitol (Sigma-aldrich) for 1 h or 0.2 M (or 0.3 M) NaCl for 3 h. Coverslips were washed twice with 1× PBS and then fixed with 4% paraformaldehyde (PFA) for 10 min at RT. After the PFA was washed, the cells were permeabilized with 0.1% Triton X-100 in 1× PBS for 10 min. After treatment with blocking buffer containing 2% BSA in PBS with 0.1% Triton X-100 for 30 min, the coverslips were incubated with primary antibody at 4°C overnight. The primary antibodies were diluted in blocking buffer; they were: polyclonal anti-TDP-43, 1∶200 (10782-2-AP, ProteinTech); monoclonal anti-G3BP, 1∶200 (611126, BD Transduction Laboratories); and monoclonal anti-phospho TDP-43 (Ser409/410), 1∶200 (MABN14, Millipore). The coverslips were then incubated with secondary antibody for 1 h at RT: goat anti-rabbit Alexa Fluor 488, 1∶1000 (A11008, Life Technologies); goat anti-mouse Alexa Fluor 555, 1∶1000 (A21422, Life Technologies); or goat anti-mouse Alexa Fluor 555, 1∶1000 (A11006, Life Technologies). The coverslips were mounted in buffer with 2.5% DABCO, 100 mM Tris-HCl (pH 8.8), 50% glycerol, and 0.2 µg/ml DAPI. Images were collected with a Zeiss AxioObserver Z1 with an Apotome imaging system.

### Co-immunoprecipitation and western blot assays

Cells were lysed in buffer containing 50 mM Tris-HCl (pH 8.0), 150 mM NaCl, 0.4 mM EDTA (pH 8.0), 1% NP-40, 0.05% sodium deoxycholate, and complete protease inhibitor cocktail (11836153001). Cell lysates were incubated with anti-HA antibody (H6908, Sigma-aldrich) overnight at 4°C before being centrifuged at 10,000× g for 10 min. Supernatant was transferred to clean tubes and incubated with protein G-Sepharose beads (17061801,GE Healthcare life sciences) for 1 h. After five washes with lysis buffer, the beads were resuspended in SDS sample buffer and boiled for 5 min before the eluted materials were subjected to standard western blot analysis: Protein samples were separated by SDS-PAGE and transferred to nitrocellulose membranes (Bio-Rad). The membranes were blocked with 5% milk in 1× phosphate-buffered saline with 0.1% Tween 20 (PBST) and incubated with the following primary antibodies: anti-c-myc- horseradish peroxidase, 1∶5000 (11814150001, Roche); monoclonal anti-c-myc, clone 9E10, 1∶3000 (M5546, Sigma-aldrich); monoclonal anti-Flag M2, clone M2, 1∶5000 (F3165, Sigma-aldrich); polyclonal anti-GAPDH,1∶30,000 (PA1-27448, Thermofisher); monoclonal anti-V5, 1∶3000 (460705, Life Technologies); polyclonal anti-TARDBP, 1∶3000 (10782-2-AP, ProteinTech), polyclonal anti-PARP, 1∶1000 (9542, Cell Signaling), polyclonal anti-Caspase 3, 1∶1000 (9662, Cell Signaling), and polyclonal anti-Cu/Zn SOD, 1∶3000 (ADI-SOD-100, Enzo life sciences). The following secondary antibodies were used: goat anti-rabbit IgG (H+L)-HRP conjugate, 1∶3000 (170–6515, Bio-Rad); goat anti-mouse IgG (H+L)-HRP conjugate, 1∶3000 (170–6516, Bio-Rad); goat anti-rabbit IgG IRDye, 1∶40,000 (680 LT, 926–68021 or 800 CW, 926–32211, LI-COR); and donkey anti-mouse IgG, 1∶40,000 (680 LT, 926–68022; or 800 CW, 926–32212, LI-COR). After incubating with secondary antibodies, the membranes were developed on films or the Odyssey image system (Li-COR).

### Cellular fractionation assays

To isolate cytoplasmic and nuclear fractions, cultured mammalian cells were harvested in 1× PBS, centrifuged at 200× g for 1 min, and washed twice with 1× PBS. The cell pellets were resuspended in a cytoplasmic extraction buffer containing 10 mM HEPES (pH 7.9), 10 mM KCl, 1.5 mM MgCl_2_, 0.1 mM EDTA, 0.5 mM DTT, 0.4% NP-40, 0.5 mM PMSF, and complete protease inhibitor cocktail, then incubated on ice for 5 min. Cell lysates were then centrifuged at 600× g at 4°C for 3 min. Supernatants were transferred to clean tubes and saved as the “cytoplasmic fraction.” The pellets were washed twice with cytoplasmic extraction buffer and centrifuged at 600× g at 4°C for 3 min. After washing, the pellets were resuspended in a nuclear extraction buffer containing 20 mM HEPES (pH 7.9), 420 mM NaCl, 1.5 mM MgCl_2_, 25% glycerol, 0.5 mM PMSF, 0.2 mM EDTA, 0.5 mM DTT, and complete protease inhibitor cocktail and vortexed at room temperature for 1 min. The resuspended samples were incubated on ice for 10 min, then vortexed for 1 min and centrifuged at 16,000× g for 10 min. The supernatants were transferred to clean tubes and saved as the “nuclear fraction.”

### Protein aggregation assays for mammalian cells and *C. elegans*

A biochemical assay was used to detect insoluble aggregated proteins according to a previously described protocol, with some modifications [60]. Mammalian cells or *C. elegans* were extracted in 200 µl of buffer containing 10 mM Tris-HCl (pH 8.0), 100 mM NaCl, 1 mM EDTA (pH 8.0), 0.5% NP-40, 50 µM iodoacetamine, and protease inhibitor (P8340, Sigma-aldrich) by using a Bioruptor ultrasonicator at 4°C for 5 min. The lysates were then transferred to an Airfuge ultracentrifuge (Beckman Coulter) and centrifuged at 25 psi (∼130,000 g) for 5 min.

The supernatant was transferred to clean tubes and saved as the “soluble” fraction. The remaining pellet was again resuspended in extraction buffer, then sonicated for 5 min. This resuspended pellet was applied to the Airfuge and centrifuged at 25 psi for 5 min. The remaining pellet was transferred to 100 µl of resuspension buffer containing 10 mM Tris-HCl (pH 8.0), 100 mM NaCl, 1 mM EDTA (pH 8.0), 0.5% NP-40, 0.5% deoxycholic acid, and 2% SDS, and sonicated for 5 min. This fraction was considered the “insoluble” protein aggregate fraction.

### Statistical analysis

Image J software was used to analyze the colocalization between TDP-43/pTDP-43 and G3BP, and the colocalization was measured by Pearson's correlative coefficient. *p* values for comparing Kaplan-Merier survival curves between groups were calculated by the Log-rank test. *p* values of qPCR, egg-laying, locomotion and Luciferase assay data were calculated with the Student's *t*-test.

## Supporting Information

Figure S1 Loss of TDP-1 improves egg-laying and locomotion defects in the absence of DAF-2. The WT control, *tdp-1(ok803lf)*, *daf-2(e1370lf)* and the *tdp-1;daf-2* double mutant were subjected to assays quantifying egg-laying (n = 10, *p<0.001) (A) and locomotion (n = 30, *p<0.05) (B). Error bars represent SEM.
(TIF)

Figure S2 DAF-16 transcriptional targets are specifically up-regulated in *tdp-1*-null *C. elegans*. (A) Loss of *tdp-1* in *C. elegans* leads to more down-regulated genes than up-regulated genes. Transcriptional profile analysis of mutant *C. elegans* lacking *tdp-1* as compared to wild-type N2 animals. There were 485 genes down-regulated (blue) and 227 genes up-regulated (red) when the threshold of -fold change (FC) was set at 1.5. (B) Quantitative RT-PCR validation of representative genes analyzed by the microarray assay (black dots) and the DAF-16 transcriptional targets (red dots).
(TIF)

Figure S3 The FHRE-Luc reporter is not affected by the transfection of TDP-43 alone. HEK293T Cells were co-transfected with Myc-TDP-43 or control Myc vector, the FHRE-Luc reporter, and the *Renilla* luciferase control. Cell lysates were subjected to dual luciferase assays, and the ratio of firefly *to Renilla* luciferase activity was used to indicate the FOXO transcriptional activity. n>3, *p*>0.05.
(TIF)

Figure S4 The dynamic change in the TDP-43 recruitment to stress granules induced by 0.2M NaCl treatment. HEK293T cells were treated with 0.2 M NaCl and analyzed at different time points by immunofluorescence microscopy for distribution of the endogenous TDP-43 (red) and the stress granule marker G3BP (green). With 30 min of the treatment, TPD-43 is predominantly nuclear. At 1 h, TDP-43 gains a slight cytoplasmic distribution in a diffuse and small punctate pattern, while G3BP is localized to well-demarcated stress granules. At 2 h, cytoplasmic TDP-43 forms larger puncta and co-localizes with G3BP in stress granules. Scale bar: 5 µM.
(TIF)

Figure S5 The dynamic change in the TDP-43 recruitment to cytoplasmic granules induced by 0.3M NaCl treatment. HEK293T cells were treated with 0.2M NaCl and analyzed at different time points by immunofluorescence microscopy for distribution of the endogenous TDP-43 (red) and the stress granule marker G3BP (green). At 1 h, TDP-43 has a somewhat diffuse and minor punctate distribution in the cytoplasm, and fewer and smaller G3BP-positive stress granules are present when compared to the 0.2M NaCl treatment. At 2 h, the TDP-43 puncta remain small and do not co-localize with G3BP-positive stress granules. Scale bar: 5 µM.
(TIF)

Figure S6 The hypotonic stress-induced translocation of TDP-43 from the nucleus to the cytoplasm is reversible. HEK293T cells were treated with 0.4 M sorbitol, 0.2 M NaCl, or 0.3 M NaCl. After 3 h of treatment, the stressors were removed, and the cells were cultured for another 24 h. Immunofluorescent staining was performed for endogenous TDP-43 (red) and the stress granule marker G3BP (green). TDP-43 is translocated back to nucleus, although G3BP-positive stress granules remain. Scale bar: 5 µM.
(TIF)

Figure S7 TDP-43 does not physically interact with FOXO proteins. HEK293T cells were co-transfected with Myc-TDP-43 and either WT Flag-FOXO3a (A) or Flag-FOXO3aAAA, which is a constitutively active form of FOXO3a (B). Co-immunoprecipitation was performed using anti-Myc (TDP-43) antibody for the pull-down, and the precipitates were analyzed by western blotting using anti-Myc-TDP-43 and anti-Flag-FOXO antibodies. No interactions between TDP-43 and the FOXO proteins were detected.
(TIF)

Figure S8 Differential cellular distribution of TDP-43 WT and mutants lacking the nuclear localization signal (ΔNLS) or the nuclear export signal (ΔNES). (A) Schematic diagrams of TDP-43 proteins with ΔNLS or ΔNES mutations. The other domains of TDP-43, including the RNA recognition domains (RRM) and the glycine-rich domain, are shown. (B) TDP-43 ΔNLS or ΔNES mutant proteins have enriched cytoplasmic or nuclear localization, respectively. HEK293T cells were transfected with the V5-TDP-43 WT, ΔNLS, or ΔNES construct. Fractionation assays were performed as described in Materials and Methods, and cytoplasmic and nuclear fractions were analyzed by western blotting. V5-TDP-43 protein was detected by an anti-V5 antibody. PARP1 and CAS3 are markers of the nuclear and cytoplasmic fractions, respectively.
(TIF)

Table S1 Primers and oligonucleotides in materials and methods.
(DOCX)
