# Supplementary figures and images for: RNA-Processing Protein TDP-43 Regulates FOXO-Dependent Protein Quality Control in Stress Response
Source: PLoS Genet. 2014 Oct 16;10(10):e1004693. doi: 10.1371/journal.pgen.1004693 (PMC4199500; doi:10.1371/journal.pgen.1004693)

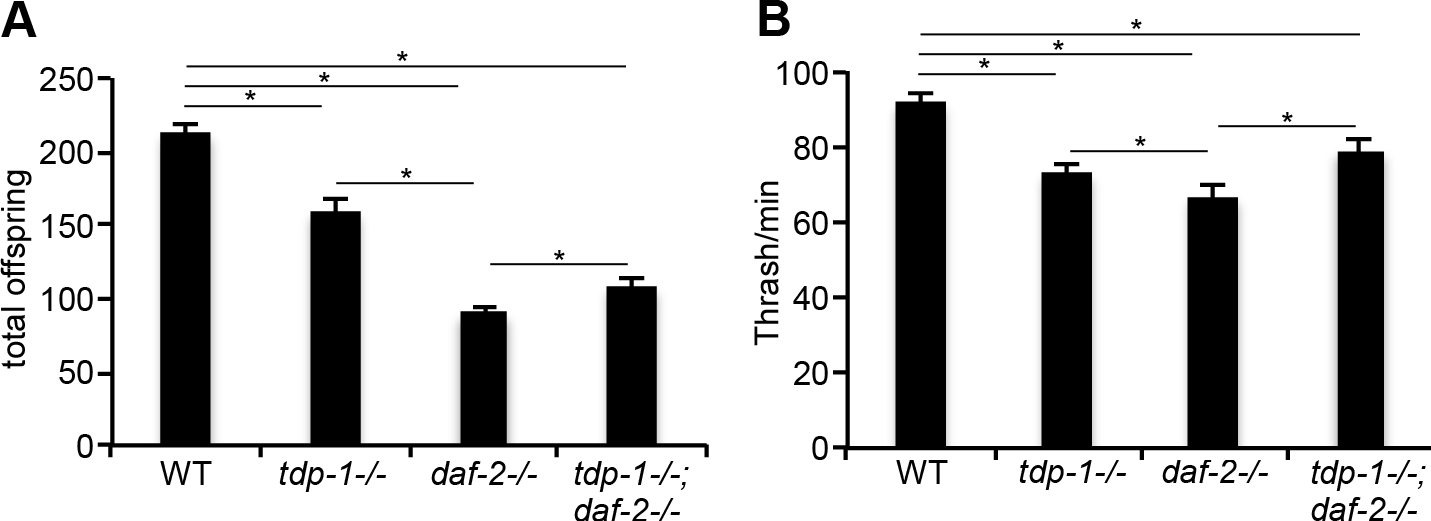

Supplement: Figure S1 — Loss of TDP-1 improves egg-laying and locomotion defects in the absence of DAF-2. The WT control, tdp-1(ok803lf), daf-2(e1370lf) and the tdp-1;daf-2 double mutant were subjected to assays quantifying egg-laying (n = 10, *p<0.001) (A) and locomotion (n = 30, *p<0.05) (B). Error bars represent SEM. (TIF) [file pgen.1004693.s001.tif]

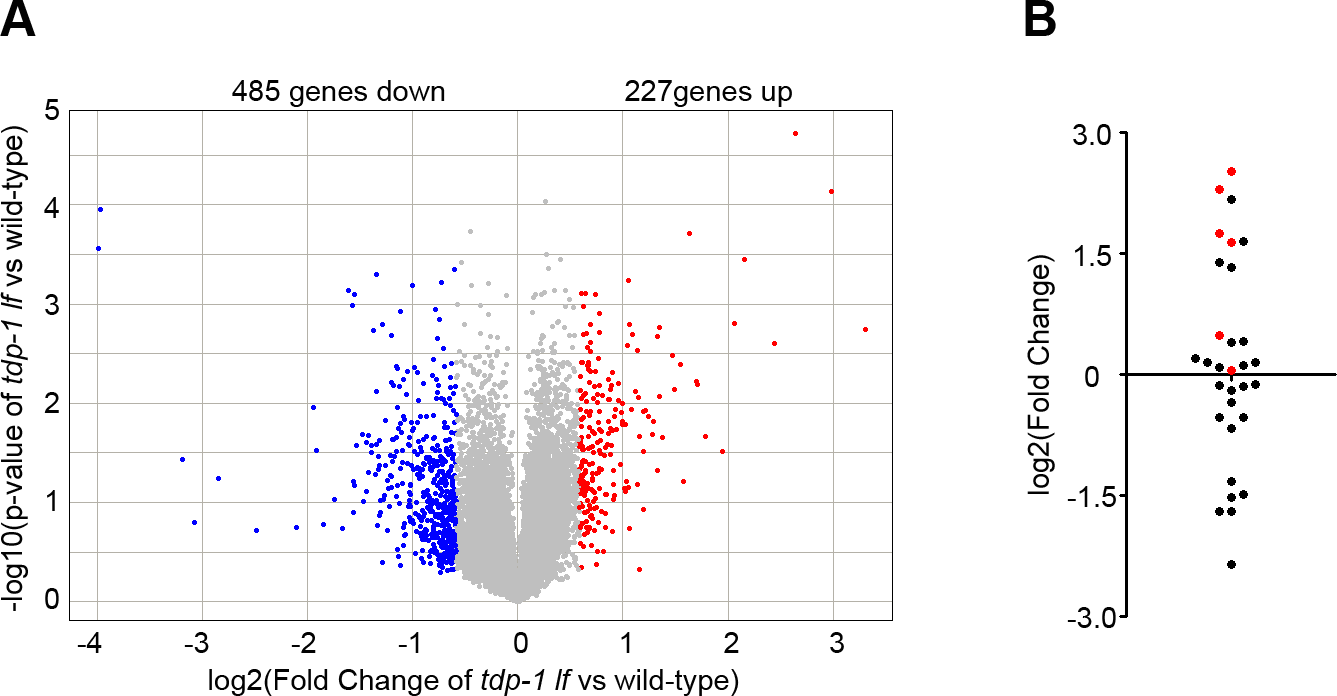

Supplement: Figure S2 — DAF-16 transcriptional targets are specifically up-regulated in tdp-1-null C. elegans. (A) Loss of tdp-1 in C. elegans leads to more down-regulated genes than up-regulated genes. Transcriptional profile analysis of mutant C. elegans lacking tdp-1 as compared to wild-type N2 animals. There were 485 genes down-regulated (blue) and 227 genes up-regulated (red) when the threshold of -fold change (FC) was set at 1.5. (B) Quantitative RT-PCR validation of representative genes analyzed by the microarray assay (black dots) and the DAF-16 transcriptional targets (red dots). (TIF) [file pgen.1004693.s002.tif]

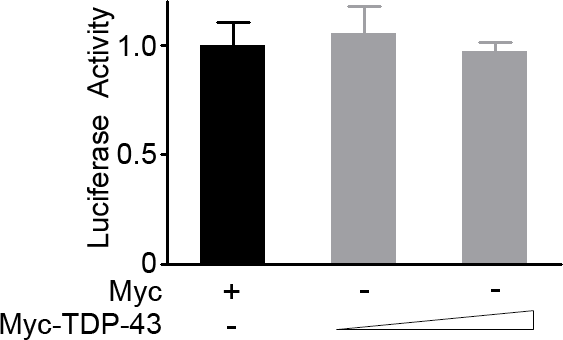

Supplement: Figure S3 — The FHRE-Luc reporter is not affected by the transfection of TDP-43 alone. HEK293T Cells were co-transfected with Myc-TDP-43 or control Myc vector, the FHRE-Luc reporter, and the Renilla luciferase control. Cell lysates were subjected to dual luciferase assays, and the ratio of firefly to Renilla luciferase activity was used to indicate the FOXO transcriptional activity. n>3, p>0.05. (TIF) [file pgen.1004693.s003.tif]

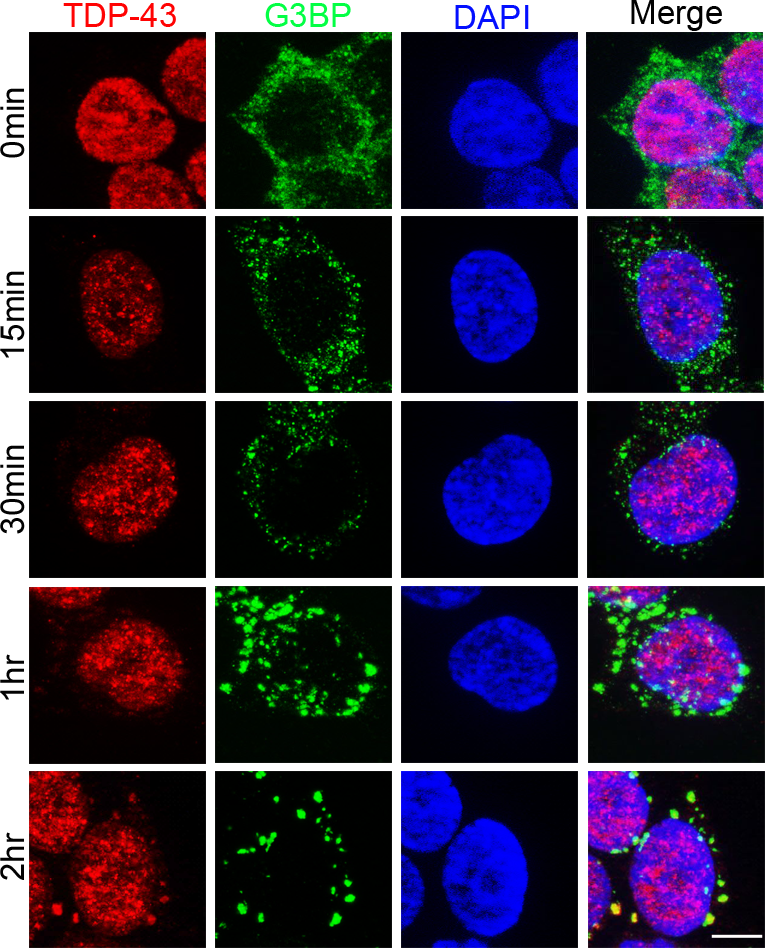

Supplement: Figure S4 — The dynamic change in the TDP-43 recruitment to stress granules induced by 0.2M NaCl treatment. HEK293T cells were treated with 0.2 M NaCl and analyzed at different time points by immunofluorescence microscopy for distribution of the endogenous TDP-43 (red) and the stress granule marker G3BP (green). With 30 min of the treatment, TPD-43 is predominantly nuclear. At 1 h, TDP-43 gains a slight cytoplasmic distribution in a diffuse and small punctate pattern, while G3BP is localized to well-demarcated stress granules. At 2 h, cytoplasmic TDP-43 forms larger puncta and co-localizes with G3BP in stress granules. Scale bar: 5 µM. (TIF) [file pgen.1004693.s004.tif]

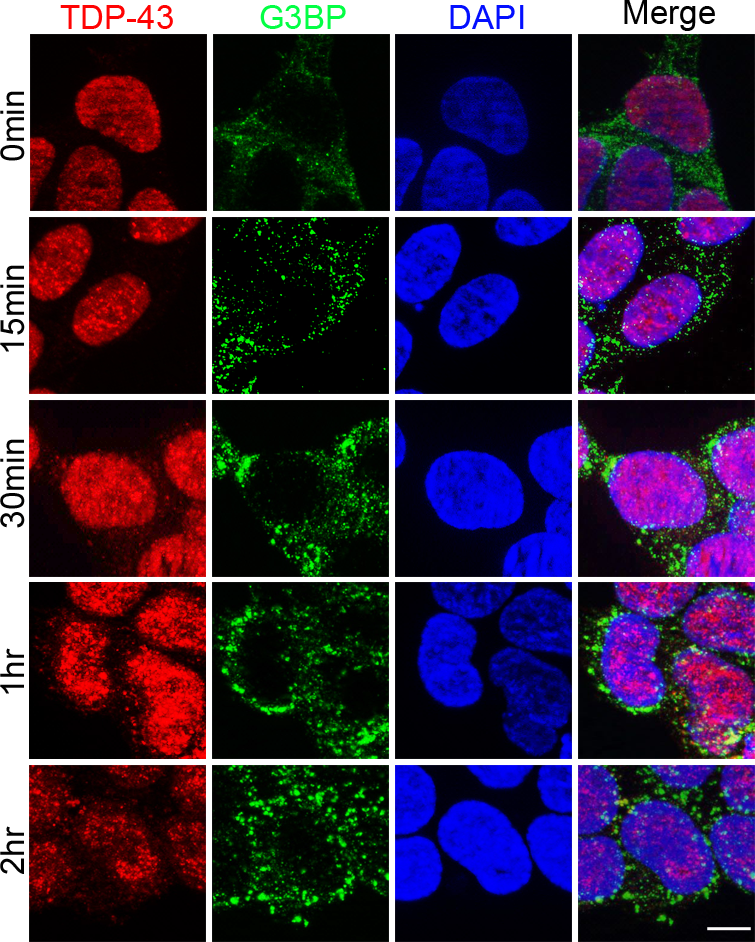

Supplement: Figure S5 — The dynamic change in the TDP-43 recruitment to cytoplasmic granules induced by 0.3M NaCl treatment. HEK293T cells were treated with 0.2M NaCl and analyzed at different time points by immunofluorescence microscopy for distribution of the endogenous TDP-43 (red) and the stress granule marker G3BP (green). At 1 h, TDP-43 has a somewhat diffuse and minor punctate distribution in the cytoplasm, and fewer and smaller G3BP-positive stress granules are present when compared to the 0.2M NaCl treatment. At 2 h, the TDP-43 puncta remain small and do not co-localize with G3BP-positive stress granules. Scale bar: 5 µM. (TIF) [file pgen.1004693.s005.tif]

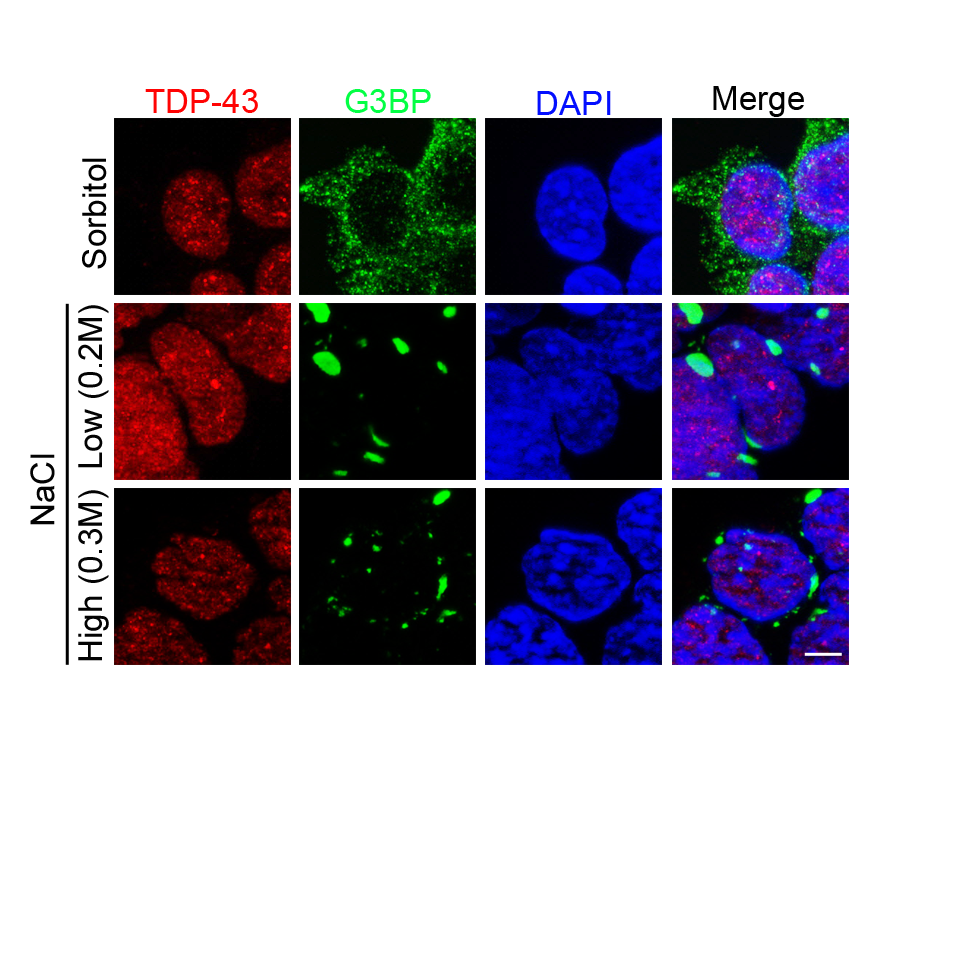

Supplement: Figure S6 — The hypotonic stress-induced translocation of TDP-43 from the nucleus to the cytoplasm is reversible. HEK293T cells were treated with 0.4 M sorbitol, 0.2 M NaCl, or 0.3 M NaCl. After 3 h of treatment, the stressors were removed, and the cells were cultured for another 24 h. Immunofluorescent staining was performed for endogenous TDP-43 (red) and the stress granule marker G3BP (green). TDP-43 is translocated back to nucleus, although G3BP-positive stress granules remain. Scale bar: 5 µM. (TIF) [file pgen.1004693.s006.tif]

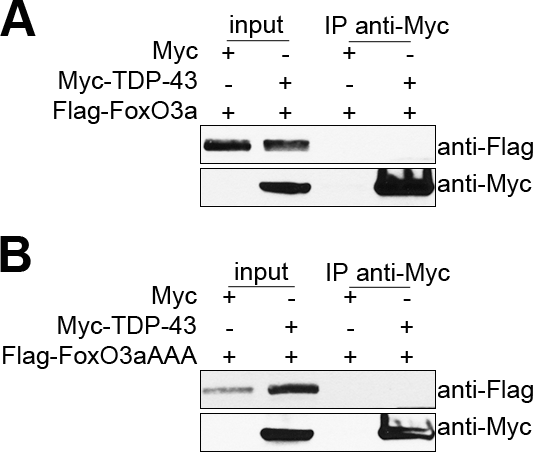

Supplement: Figure S7 — TDP-43 does not physically interact with FOXO proteins. HEK293T cells were co-transfected with Myc-TDP-43 and either WT Flag-FOXO3a (A) or Flag-FOXO3aAAA, which is a constitutively active form of FOXO3a (B). Co-immunoprecipitation was performed using anti-Myc (TDP-43) antibody for the pull-down, and the precipitates were analyzed by western blotting using anti-Myc-TDP-43 and anti-Flag-FOXO antibodies. No interactions between TDP-43 and the FOXO proteins were detected. (TIF) [file pgen.1004693.s007.tif]

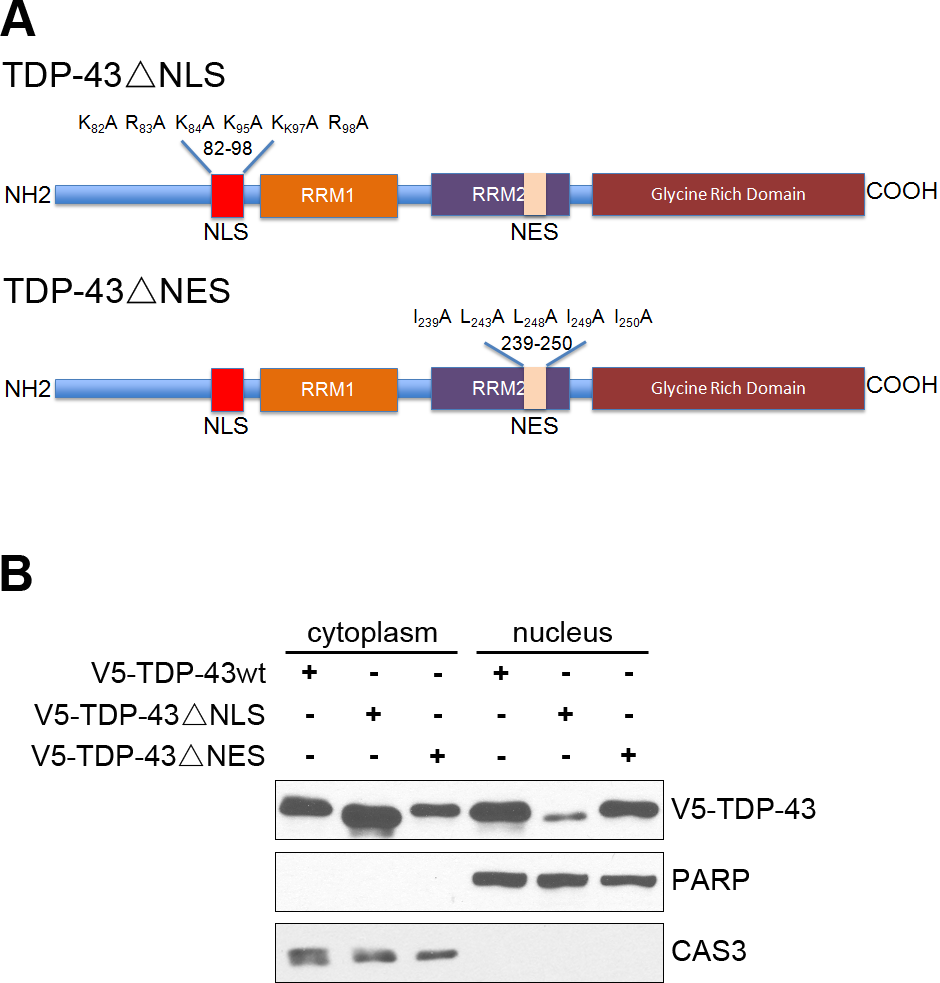

Supplement: Figure S8 — Differential cellular distribution of TDP-43 WT and mutants lacking the nuclear localization signal (ΔNLS) or the nuclear export signal (ΔNES). (A) Schematic diagrams of TDP-43 proteins with ΔNLS or ΔNES mutations. The other domains of TDP-43, including the RNA recognition domains (RRM) and the glycine-rich domain, are shown. (B) TDP-43 ΔNLS or ΔNES mutant proteins have enriched cytoplasmic or nuclear localization, respectively. HEK293T cells were transfected with the V5-TDP-43 WT, ΔNLS, or ΔNES construct. Fractionation assays were performed as described in Materials and Methods, and cytoplasmic and nuclear fractions were analyzed by western blotting. V5-TDP-43 protein was detected by an anti-V5 antibody. PARP1 and CAS3 are markers of the nuclear and cytoplasmic fractions, respectively. (TIF) [file pgen.1004693.s008.tif]
