# Supplementary material for: RNA-Processing Protein TDP-43 Regulates FOXO-Dependent Protein Quality Control in Stress Response
Source: PLoS Genet. 2014 Oct 16;10(10):e1004693. doi: 10.1371/journal.pgen.1004693 (PMC4199500; doi:10.1371/journal.pgen.1004693)
Supplement: Table S1 — Primers and oligonucleotides in materials and methods. (DOCX) [file pgen.1004693.s009.docx]

| **Table S1. Primers and oligonucleotides in materials and methods.** | | |
| --- | --- | --- |
| **Gene Name** | **Sequences** | |
| *gdh-1* (qPCR) | forward | CGGTATCATCGAAGGACT |
|  | reverse | GTCCATCTCTCCACAGCT |
| *mtl-1* (qPCR) | forward | CAATTTGACTGCTGAAAT |
|  | reverse | [TACTTCTCACAACACTTG](about:blank) |
| *mtl-2* (qPCR) | forward | GGAACTAAAGATTGCGATTG |
|  | reverse | GCAGCATTTCTTCTCACT |
| *dct-17* (qPCR) | forward | CGCAACTTACACATACATT |
|  | reverse | CAAGACTACCTGATCGTTA |
| *dct-8* (qPCR) | forward | TTCCTTCGCAACCTCATT |
|  | reverse | TAGTCCCAGTCCAGTGTT |
| *dao-4* (qPCR) | forward | CACTGTATTCTATTCTTCTCATT |
|  | reverse | TGTAACCTTGCTCGAATT |
| *F44D12.4* (qPCR) | forward | ATTACCAAGTTGCTCGTA |
|  | reverse | TAGTCATCTTCATAGTTCCT |
| *tdp-43* (qPCR) | forward | GCCTTCGGTTCTGGAAATAACTC |
|  | reverse | CCCGACCCTGCATTGGAT |
| pRK5-myc-TDP-43  primers | forward | GATCCTCGAGATGTCTGAATATATTCGGGTAACCG (XhoI) |
|  | reverse | ATG ATCGCTGAGCCATTCCCCaGCCAGAAGACTTAG (XhoI) |
| TDP-43 Gateway (pDonor-221) primers | forward | GGGGACAAGTTTGTACAAAAAAAGCAGGCTTCGAAGGAGATAGAACCATGTCTGAATATATTCGGG |
|  | reverse | GGGGACCACTTTGTACAAGAAAGCTGGGTCCTACATTCCCCAGCCAGAAGACTTAGAATCCATGCT |
| TDP-43 shRNA oligo | (i) | AGTTGTCTCAAGTCAAATGGATTCATCAC |
|  | (ii) | AATATGAAGCCTTCATTTAATCTCTGCA |
|  | (iii) | TATTCTGCCATAGGAATACTGTCTACATG |
| Control shRNA oligo | GCACTACCAGAGCTAACTCAGATAGTACT | |
